# Supplementary figures and images for: A Specific Mixture of Propolis and Carnosic Acid Triggers a Strong Fungicidal Action against Cryptococcus neoformans
Source: Antibiotics (Basel). 2021 Nov 13;10(11):1395. doi: 10.3390/antibiotics10111395 (PMC8614879; doi:10.3390/antibiotics10111395)

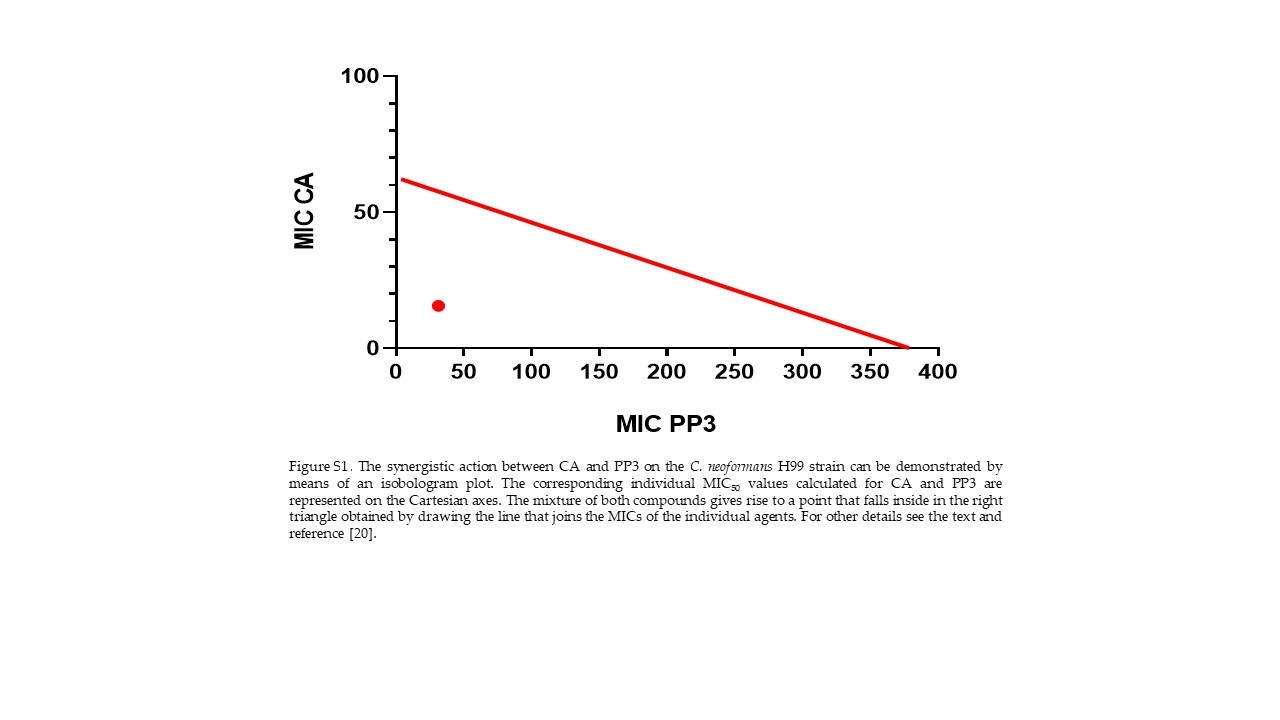

Supplement: Supplementary file 1 [file antibiotics-10-01395-s001.zip › antibiotics-1448034-supplementary.jpg]
